# ^99m^Tc-MIBI Washout Rate to Evaluate the Effects of Steroid Therapy in Cardiac Sarcoidosis.

**Published:** 2013

**Authors:** Masayoshi Sarai, Sadako Motoyama, Yasuchika Kato, Hideki Kawai, Hajime Ito, Kayoko Takada, Ryuji Yoda, Hiroshi Toyama, Shin-ichiro Morimoto, Yukio Ozaki

**Affiliations:** 1 Department of Cardiology, Fujita Health University School of Medicine; 2 Department of Radiology, Fujita Health University School of Medicine

**Keywords:** ^99m^tc-MIBI Scintigraphy, Washout Rate, Steroid Therapy, Cardiac Function, Cardiac Sarcoidosis

## Abstract

**Objective:**

We sought to determine the usefulness of the ^99m^Tc-MIBI (MIBI) washout rate for the evaluation of steroid therapy in cardiac sarcoidosis (CS).

**Methods:**

Eleven CS patients underwent MIBI myocardial SPECT both before and 6 months after initiating steroid therapy. The washout rate (WOR) of MIBI was calculated using early and delayed polar map images. The washout score (WOS) of MIBI was derived from the difference between the early and delayed total defect scores (TDS).

**Results:**

Serum ACE and BNP exhibited significant improvement after the therapy (*p* = 0.004, *p* = 0.045). In the LV function, EDV and E/A ratio exhibited significant improvement after the therapy (*p* = 0.041, *p* = 0.007), while there were no significant differences between before and after therapy in EF or ESV. Early and delayed TDS showed no significant differences between before and after the therapy. In contrast, WOR differed significantly (p <. 0001), while WOS did not differ significantly between before and after the therapy.

**Conclusion:**

The washout rate of MIBI is suitable for assessment of cardiac function in CS with steroid therapy, being especially better than the washout score of MIBI for assessment of disease activity of mild myocardial damage in CS with steroid therapy.

## Introduction

Sarcoidosis is a systemic granulomatous disorder whose prognosis is generally favorable (1). However, cardiac involvement may carry a poor prognosis (2). For this reason, it is essential to determine whether such cardiac lesions are present. Also, when considering whether to administer steroid therapy, the earliest possible diagnosis is important, although patients with cardiac sarcoidosis (CS) may be misdiagnosed in part because of the low rate of diagnostic success achieved by endomyocardial biopsy (3). Regarding the imaging diagnosis, 67-Ga scintigraphy and 201-Tl myocardial perfusion single-photon emission computed tomography (SPECT) have been widely employed.

In 2006, the Japanese Society of Sarcoidosis and Granulomatous Disorders revised the former 1993 guidelines for the diagnosis of Cardiac sarcoidosis (4). Criteria on new imaging modalities, such as cardiac MRI and 18F-FDG PET

were included in these guidelines. Although there are many articles on the diagnosis of CS by these imaging equipments, there are few reports on predicting the efficacy of steroid therapy.

In nuclear cardiology, reverse redistribution of ^99m^Tc-MIBI (MIBI) has been noted after direct percutaneous coronary intervention in patients with acute myocardial infarction (5). These observations suggested that the elimination of MIBI can be used to estimate ongoing myocardial damage. Moreover, the myocardial washout of MIBI is increased in patients with heart failure (6, 7) and various cardiomyopathies (8).

In this study, we investigated the usefulness of MIBI washout rate for the evaluation of steroid therapy in patients with CS.

## Materials and Methods

### 

#### Study Population

Eleven patients who had been diagnosed with CS (2 men, 9 women, mean age 68 ± 4 years) and received steroid therapy in our institution were analyzed retrospectively (Table 1). We diagnosed patients with CS according to the guidelines for the diagnosis of cardiac sarcoidosis of the Japanese Ministry of Health, Labor and Welfare (4). All patients had received prednisone 30 mg per day, which was then tapered gradually until a maintenance dose was reached.

**Table 1:** Baseline cilinical characterists.

Case	Age (yrs]	Sex	ECG	BP (mmHg)	HR (bpm)	BUL	IVS thinness	other organs	^67^Ga Sintigfaphy	EMB	Pre ACE (IU/L)	Post ACE (IU/L)	Pre BNP (pg/ml)	Post BNP (pg/ml)	Pre EF (%)	Post EF (%)	Pre EDV (ml)	Pre ESV (ml)	Post EDV (ml)	Post ESV (ml)	Pre E/A ratio	Post E/A ratio	Pre TDS early	Pre TDS delay ed	Post TDS early	Post TDS delayed	Pre WOS	Post WOS	Pre WOR (%)	Post WOR (%)	drugs
1	69	F	CAVB(PM), AF	87/61	70	N	P	eye	Nene	N	25.9	10.4	1500	556	34	34	188	124	159	106	0.99	1.15	9	9	9	9	0	0	21.2	18.2	ARB, BB, D
2	73	F	CAVB(PM), CRBBB	104/66	90	N	P	LN	htart, LN	P	16.2	4.6	98	37	89	91	48	5	37	3	0.85	0.93	0	0	0	0	0	0	26.7	18.7	None
3	72	M	CAVB(PM), AF	112/70	80	N	N	eye, muscle	heart	N	25.3	8.6	89	25	63	78	66	24	61	13	1.06	1.13	3	3	3	3	0	0	29.7	13.8	ARB, CCB, D
4	65	F	CAVB(PM)	104/72	82	N	N	LN, lung	heart, LN, lang	NP	23.5	15.7	102	27	65	67	83	29	80	26	0.69	1.16	6	6	8	8	0	0	21.8	13.6	None
5	71	F	CRBBB	96/52	74	P	N	skin	heart	N	2.6	0.7	527	515	40	48	157	94	93	49	0.88	1.21	18	17	11	13	-1	2	31.7	21.8	ARB
6	63	F	CAVB(PM)	98/56	52	P	N	LN, eye	LN	P	2.5	9.1	203	39	64	77	65	23	51	12	0.84	1.03	4	5	3	3	1	0	21.5	14.9	ARB, D
7	73	F	CAVB(PM)	140/60	96	P	P	LN, skill, muscle	heart, LN, skin, mustie	N	22.5	7.1	121	80	55	56	90	41	81	36	0.97	1.23	7	7	5	5	0	0	21.8	14.6	None
8	62	F	CRBBB	94/56	BS	N	N	LM. skin, muscle	LN, skir, muscle	N	31.9	7.0	611	198	49	43	99	51	84	47	0.88	1.29	12	11	10	10	-1	0	29.9	23.3	ARB, D
9	62	M	CAVB(PM)	108/62	68	N	N	LN, eye	LN	N	15.6	9.0	390	152	41	34	114	68	104	68	1.04	1.18	4	4	4	4	0	0	28.3	2.3	ARB, BB, D
10	72	F	CAVB(PM)	98/62	70	N	P	LN	LN	N	7.2	6.7	115	53	43	30	124	70	126	88	0.94	1.08	13	13	12	12	0	0	26.8	18.7	ARB.D
11	72	F	CAVB(PM)	168/62	50	P	N	LN	LN	NP	21.2	7.8	63	38	89	77	38	4	46	11	1.13	0.94	0	0	0	0	0	0	16.8	4.3	ARB, CCB, D

*ECG*electronicardiography, *BP* blood pressure, *HR* heart rate, *BHL* bilateral hillar lymphadenopathy, *IVS* interventricular septum, *EMP* endomyocardial biopsy, *ACE*angiotensin-converting enzyme, *BNP* brain natriuretic peptide, *EF* ejection fraction, *EDV* end systolic volume, *E/A* ratio peak early diastole/late diastole transmitral flow velocity ratio, *TDS* total defect score, *WOS* washout score, *WOR* washout rate, *CAVB* complete atrioventricular block, *PM* pacemaker, *AF*atrial fibrillation, *CRBBB* complete right bundle branch block *N* negative, *P* positive, *LN* lymph node, *NP* not performed, *ARB* angiotensin receptor blocker, *BB* beta blocker, *CCB* calcium channel blocker, *D* diuretic.

#### Study Protocol

The study protocol was approved by the ethics committee of our institution. Venous blood samples were obtained for serum B-type natriuretic peptide (BNP) and serum angiotensin converting enzyme (ACE) before and 6 months after the start of steroid therapy. All patients underwent MIBI myocardial SPECT and echocardiography before and 6 months after initiating this therapy.

#### MIBI myocardial SPECT

In each patient, 600 MBq of MIBI (FUJIFILM RI Pharma Co. Ltd, Tokyo, Japan) was intravenously injected under resting conditions. SPECT imaging data were acquired at 1 h (early imaging) and 4 h (delayed imaging) after injection, using a dual-head gamma camera (Vertex plus; Philips/ADAC Laboratories, Bothell, WA, USA). We used a vertex general purpose (VXGP) collimator with energy window at 20% and photo-peak at 140 keV. All images were acquired with ECG gating. SPECT imaging data were acquired using a 90 degree rotation arc, 32 projections (40 s/projection), 16 frames per heart cycle, and 64 × 64 matrices. After processing the projection images with a Butterworth filter (cut-off frequency 0.45 cycles/cm, cut-off order 5), reconstructive processing was performed with a Ramp filter without correction for attenuation or scatter.

The left ventricular end-diastolic volume (LVEDV), left ventricular end-systolic volume, and left ventricular ejection fraction (LVEF) were calculated from gated SPECT data of early images with a quantitative ECG-gated SPECT program (QGS, Cedars-Sinai Medical Center, Los Angeles, California, USA).

The SPECT short axis slices of MIBI were assembled in polar map images to assess the regional distribution myocardial tracer uptake. After correcting for physical decay of technetium 99m, the washout rate (WOR) was calculated using the following equation: [(early counts – delayed counts) / (early counts)] ×100 (%). SPECT images were scored visually using the 17-segment model of the left ventricle with a 5-point scoring system (0 = normal uptake, 1 = mildly reduced, 2 = moderately reduced, 3 = severely reduced, and 4 = no uptake). Total defect scores (TDS) were calculated by adding the scores of 17 segments on the individual early and delayed images, respectively. The washout score (WOS) was considered as the difference between the early TDS and delayed TDS.

#### Echocardiography

Echocardiography was performed using commercially available Doppler echocardio-graphy unit (Philips Sonos 7500) with a 1.5-3.5 MHz duplex imaging transducer. Patients were studied in a left lateral decubitus. Peak velocity of early diastolic filling of mitral inflow (E) and late diastolic filling due to atrial contraction (A) were recorded appropriately and then E/A ratio was calculated as diastolic functional index respectively.

#### Statistical analysis

All values are presented as mean ± standard deviation (SD). Differences were assessed for statistical significance using the paired *t*-test. The differences were considered significant when *p* < 0.05.

## Results

All patients exhibited some ECG abnormalities. Eight patients had extracardiac lesions, and cardiac involvement was observed in the endomyocardial biopsy in only 2 patients (18%) (Table 1). Serum ACE and BNP improved significantly after the therapy (17.7 ± 9.9 vs 7.9 ± 3.7 IU/L, *p* = 0.004, 347 ± 428 vs 156 ± 196 pg/ml, *p* = 0.045). In the LV function, EDV decreased significantly after the therapy (97 ± 46 vs 84 ± 36 ml, *p* = 0.041) and there were no significant differences between before and after the therapy in EF or ESV (57 ± 19 vs 58 ± 21 %, ns, 48 ± 38 vs 42 ± 34 ml, ns). E/A ratio increased significantly after the therapy (0.93 ± 0.12 vs 1.19 ± 0.24, *p* = 0.007). In the SPECT data, early and delayed TDS showed no significant differences between before and after the therapy (6.9 ± 5.6 vs 5.9 ± 4.3, ns, 6.8 ± 5.3 vs 6.1 ± 4.6, ns). In the washout analysis, WOR differed significantly between before and after therapy (25 ± 5 vs 17 ± 5 %, *p* <. 0001), WOS did not differ significantly between before and after the therapy (-0.1 ± 0.5 vs 0.2 ± 0.6, ns) (Table 2).

**Table 2:** Changes in biomarkers, cardiac function and TDS/WOS/WOR.

	Pre treatment	Post treatment
**ACE (IU/L)**	17.7 ± 9.9	7.8 ± 3.7*
**BNP (pg/ml)**	347 ±428	156 ± 196**
**EF (%)**	57 ± 19	58 ± 21
**EDV (ml)**	97 ± 46	84 ± 36**
**ESV (ml)**	48 ± 38	42 ± 34
**E/A ratio**	0.93 ± 0.12	1.19 ± 0.24*
**TDS early**	6.9 ± 5.6	5.9 ± 4.3
**TDS delay**	6.8 ± 5.3	6.1 ± 4.6
**WOS**	-0.1 ± 0.5	0.2 ± 0.6
**WOR (%)**	25 ± 5	17 ± 5*

*ACE* angiotensin-converting enzyme, *BNP* brain natriuretic peptide, *EF* ejection fraction.

*EDV* end diastolic volume, *ESV* end systolic volume, *E/A ratio* peak early diastole/late diastole transmitral flow velocity ratio.

*TDS* total defect score, *WOS* washout score, *WOR* washout rate

* *p* < 0.01 versus the pre treatment phase

** *p* < 0.05 versus the pre treatment phase

## Case presentation

**Figure 1:**
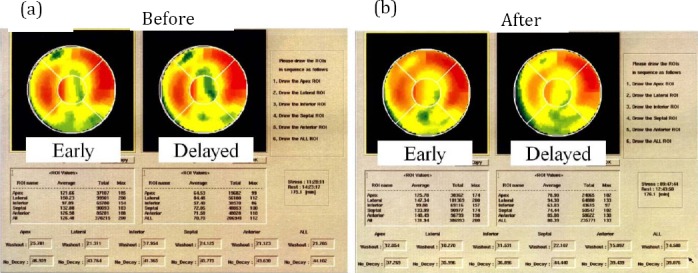
a) Polar map SPECT images of a 73–year-old woman (case 7). MIBI uptake decreased in the anterior, apical and inferior wall in the early images of SPECT before therapy. However, in the delayed images, no visual washout was observed, but WOR was high. b) After therapy, polar map SPECT images were improved slightly, however, no visual washout was observed, but WOR was improved (13.6%). LVEF, LVEDV and LVESV were not changed. Moreover, E/A ratio was normalized. ACE and BNP were also normalized.

Case 7 [a 73-year old woman]: In the early images of SPECT before initiating steroid therapy, MIBI uptake was decreased in the anterior, apical and inferior wall. Then, in the delayed images, the uptake was almost same. In this case, early TDS was 7 and WOS was 0, but WOR was increased (21.8%). In LV functional analysis, LVEF, LVEDV and LVESV was normal, but E/A ratio was low. Moreover, ACE and BNP was elevated. Six month after initiating steroid therapy, the SPECT images were improved slightly (early TDS: 5), but WOS was 0 and WOR was improved (14.6%). LVEF, LVEDV and LVESV were not changed. E/A was normalized. ACE and BNP were also normalized. (Figure 1a, b)

## Discussion

Sarcoidosis is a systemic granulomatous inflammatory disease of unknown etiology. Early diagnosis and initiation of treatment of cardiac sarcoidosis are essential because cardiac involvement, especially the severity of heart failure and ventricular remodeling, is an important prognostic factor. Starting steroid therapy before the occurrence of systolic dysfunction results in an excellent clinical outcome (9). Previous studies have focused on the detection of active inflammation in patients with CS using ^18^F-FDG PET scintigraphy (10, 11), but there are few reports on predicting the responsiveness to steroid therapy (12). Both the identification of cardiac involvement of sarcoidosis and the assessment of CS cardiac function are very important for the management of CS.

Myocellular uptake and retention of MIBI are strongly dependent on mitochondrial and plasma membrane potentials both qualitatively and quantitatively (13). Irreversible cellular injury induced by a cytochrome-*c* oxidase inhibitor or a sarcolemmal membrane detergent increased the clearance of MIBI in cultured chick embryo ventricular myocytes (14). Previous studies showed that quantitative global assessment of the MIBI washout rate is useful for evaluating global myocardial damage in patients with heart failure (6-8). But no study has focused on the usefulness of the MIBI washout rate for assessment of cardiac sarcoidosis.

In the present study, WOR differed significantly between before and after the initiation of steroid therapy. We noted a significant decrease in ACE, BNP, EDV and WOR and increase in E/A ratio, but no significant changes in ESV, LVEF, TDS (early and delayed) or WOS. Kudoh *et al* (12) reported that regional myocardial washout of ^99m^Tc-tetrofosmin correlated with the LV functional recovery after the initiation of steroid therapy in CS patients. They used semiquantitative regional visual scoring as an index of TF washout, but found no significant difference in WOS between before and after steroid therapy, in contrast to significant decrease of EDV, ESV and TDS (initial and delayed) and increase of LVEF.

Previous studies have demonstrated a significant prevalence of diastolic dysfunction in patients with pulmonary sarcoidosis (15) and that the increased ^99m^Tc-sestamibi washout is associated with impairment in prolonged myocardial relaxation in patients with hypertrophic cardiomyopathy (16). Iwanaga *et al* (17) reported that plasma BNP levels reflect left ventricular end-diastolic wall stress, not only in patients with systolic heart failure, but also in those with diastolic heart failure. In the present study, E/A ratio and WOR differed significantly between before and after steroid therapy. This result showed improvement of diastolic function in CS with steroid therapy and WOR is possible to represent left ventricular functional recovery especially diastolic functional recovery in CS with steroid therapy.

Sarcoid granulomas show a localized distribution within the myocardium on pathological examination (3). This is the reason why regional visual scoring as an index of washout of ^99m^Tc-tetrofosmin is suitable for evaluation of the myocardial damage caused by sarcoidosis. But, it is impossible for WOS to evaluate washout in CS without regional washout change. In such cases, quantitative global assessment of the MIBI washout rate might be more useful than regional visual washout score such as WOS.

This study has some limitations. Firstly, small numbers of patients in a single center were enrolled and were observed retrospectively. Secondly, we have not investigated the between an increased washout rate of MIBI and pathological findings of CS. The mechanism controlling the MIBI washout of CS was not clarified. Finally, MIBI washout may be affected by both inflammation and perfusion of myocardium in CS. In this study, we could not confirm which factor affect MIBI washout.

## Conclusions

The present study showed that the washout rate of MIBI is suitable for assessment of CS cardiac function with steroid therapy. Especially, the washout rate of MIBI is better than regional washout score for assessment of left ventricular diastolic functional recovery in CS with steroid therapy.
